# M-opsin protein degradation is inhibited by MG-132 in *Rpe65*^−/−^ retinal explant culture

**Published:** 2012-06-13

**Authors:** Kota Sato, Taku Ozaki, Sei-ichi Ishiguro, Mitsuru Nakazawa

**Affiliations:** 1 Department of Ophthalmology, Hirosaki University Graduate School of Medicine, Hirosaki, Aomori, Japan; 2 Department of Biochemistry and Biotechnology, Division of Cell Technology, Hirosaki University Faculty of Agriculture and Life Science, Hirosaki, Aomori, Japan

## Abstract

**Purpose:**

The 65 kDa retinal pigment epithelium-specific protein, RPE65, is an essential enzyme for 11-*cis*-retinal synthesis in the eye. Mutations of the *RPE65* gene in humans result in severe vision loss, and *Rpe65*^−/−^ mice show early cone photoreceptor degeneration. We used an explant culture system to evaluate whether posttranslational downregulation of M-opsin protein in *Rpe65*^−/−^ mice is caused by proteolytic degradation.

**Methods:**

The eyes of three-week-old *Rpe65*^−/−^ mice were incubated in culture medium. Western blot analysis was used to evaluate the level of M-opsin protein, and immunofluorescence was used for protein localization. The transcriptional level of M-opsin was evaluated with real-time reverse-transcriptase-PCR.

**Results:**

Degradation of the M-opsin protein in *Rpe65*^−/−^ mouse retina was inhibited by the proteasome inhibitor MG-132 but not by the lysosomal inhibitor pepstatin A and E64d. 9-*cis*-retinal, used as an analog of 11-*cis*-retinal, increased M-opsin protein but did not increase M-opsin mRNA. Moreover, 9-*cis*-retinal did not change the transcriptional levels of photoreceptor specific genes.

**Conclusions:**

Our data suggest that M-opsin protein was degraded through a proteasome pathway and that M-opsin degradation was suppressed with 9-*cis*-retinal treatment in *Rpe65*^−/−^ mice to some extent.

## Introduction

Photon absorption initiates phototransduction in retinal photoreceptors and is essential for vision in the eye. Absorption occurs via the chromophore 11-*cis*-retinal binding apo-protein, opsin [1]. After absorption of a photon, 11-*cis*-retinal is isomerized to all-*trans*-retinal [2] and regenerated by a series of retinoid metabolism transformations of enzymatic reactions called the retinoid cycle [3].

The retinal pigment epithelium-specific protein 65 kDa (RPE65) has been identified as the catalyst for the isomerization of all-trans-retinyl esters, which is converted from all-trans-retinol (vitamin A) by lecithin-retinol acyltransferase (LRAT), to 11-cis-retinol in the visual cycle pathway [4-7]. RPE65 plays a critical role in the production of 11-*cis*-retinal; *Rpe65*^−/−^ mice cannot generate 11-*cis*-retinal [7]. Measurement of visual function with electroretinogram (ERG) in *Rpe65*^−/−^ mice showed almost no response [8-10]. Photoreceptors have been described as degenerated in *Rpe65*^−/−^ mice and *Rpe65* mutant mice [11-14] like other models of retinitis pigmentosa [15]. Further, mutation of the *RPE65* gene is the cause of Leber congenital amaurosis Type 2 and retinitis pigmentosa [16-19]. These reports indicate that 11-*cis*-retinal synthesis by RPE65 is essential for maintaining photoreceptor cells and visual function in mouse and human retinas.

Moreover, 11-*cis*-retinal and other retinoids have potential as pharmacological chaperones. Opsin stability is increased by binding 11-*cis*-retinal in vitro [20-22]. We previously reported that M-opsin mRNA levels in *Rpe65*^−/−^ mice at three and five weeks of age were not significantly different from those of wild-type mice. In contrast, M-opsin protein expression was at a low level [23]. These observations suggest that M-opsin protein has decreased stability and undergoes proteolysis in *Rpe65*^−/−^ mice lacking 11-*cis*-retinal. Znoiko et al. [11] previously reported, however, that M-opsin mRNA was downregulated in *Rpe65*^−/−^ mice at early ages. Recently, Zhang et al. [24] reported M-opsin protein was markedly reduced in gene encoding LRAT (*Lrat*^−/−^) mice whereas the S-opsin level remained the same compared with wild-type mice. The results also suggested that M-opsin degradation occurred posttranslationally in the absence of a chromophore, 11-*cis*-retinal.

In the current study, we evaluated whether posttranslational downregulation of M-opsin protein in *Rpe65*^−/−^ mice was caused by proteolytic degradation. To characterize M-opsin degradation, we used three inhibitors: pepstatin A (aspartic protease inhibitor), E64d (cysteine protease inhibitor), and MG-132 (proteasome inhibitor). Additionally, we evaluated whether posttranslational downregulation of M-opsin protein in *Rpe65*^−/−^ mice was caused by 11-*cis*-retinal depletion. Instead of 11-*cis*-retinal, we used the chromophore 9-*cis*-retinal because 9-*cis*-retinal also binds with apo-opsins to form functional pigments and is known to be relatively stable as an analog to 11-*cis*-retinal [8,25]. The present study focused on determining the degradation pathway of M-opsin protein in *Rpe65*^−/−^ mice and whether the degradation was prevented by 9-*cis*-retinal.

## Methods

### Animals

All experimental procedures were designed to conform ethically to the Association for Research in Vision and Ophthalmology (ARVO) Statement for Use of Animals in Ophthalmic Vision Research and Hirosaki University Guidelines for Animals in Research. *Rpe65*^−/−^ mice were generously provided by Matthew M. LaVail (University of California, San Francisco, CA) and T. Michael Redmond (National Eye Institute, Bethesda, MD). C57BL/6 mice were obtained from Clea (Tokyo, Japan) and used as wild-type animals. Animals were maintained in the Hirosaki University Graduate School of Medicine Animal Care Service Facility under a cycle of 12-h light (50 lux illumination in the cage) and 12-h dark (<10 lux of environmental illumination). Experiments were performed on three-week-old mice.

### Explant culture

Eyes of wild-type and *Rpe65*^−/−^ mice were enucleated, and the corneas were removed on ice under the stereo microscope. The superior edges of eyecups were partially cut with a surgical knife for orientation. The eyecups were then rinsed with Hanks’ Balanced Saline Solution (HBSS; Nissui Pharmaceutical, Tokyo, Japan) and incubated in culture medium. Culture medium was a composite made of 50% Dulbecco’s modified Eagle’s Medium (DMEM; Nissui Pharmaceutical), 25% fetal bovine serum, and 25% HBSS supplemented with 2 mM L-glutamine, 5.75 mg/ml glucose, and antibiotics (100 μg/ml streptomycin and 100 units/ml penicillin; Wako Pure Chemical Industries, Osaka, Japan) according to the Osakada et al. [26] modification. Four eyes were incubated in 3 ml culture medium in each well with 6-well plates purchased from Nunc^TM^ (Thermo Fisher Scientific, Roskllide, Denmark) and maintained in a humidified atmosphere of 5% CO_2_ and 95% air at 37 °C. Pepstatin A, E64d (Peptide institute, Osaka, Japan), and MG-132 (Sigma-Aldrich, St. Louis, MO) were dissolved with a final concentration of 1% dimethyl sulfoxide (DMSO; Wako), and 9-*cis*-retinal (Sigma-Aldrich) was dissolved with 1% ethanol (Wako). After incubation with protease inhibitors or 9-*cis*-retinal, retina-RPE choroids were collected for western blotting and for real-time reverse-transcriptase polymerase chain reaction (RT–PCR), and the eyecups were used for the cryosections. The explant culture was performed in a CO_2_ incubator under dark conditions.

### Preparation of retinal sections

After treatment with protease inhibitors or 9-*cis*-retinal, eyecups were first embedded in OCT compound (Sakura, Tokyo, Japan), and then cryosections were made at a thickness of 5 μm in cryostat. Sections were fixed with 4% paraformaldehyde in phosphate-buffer (PB; pH 7.4) for 15 min before various staining experiments. The superior hemispheres of the eyecups were employed for subsequent experiments.

### Hematoxylin and eosin staining

Hematoxylin staining was performed with Carrazzi’s hematoxylin solution (Muto Pure Chemicals, Tokyo, Japan) for 5 min. After a wash with running water, eosin staining was performed with 1% eosin Y solution (Muto Pure Chemicals) for 1 min. The sections were washed and dehydrated with ethanol of serial dilution and enclosed with MGK-S (Matsunami Glass, Osaka, Japan).

### Terminal deoxynucleotidyl transferase-mediated dUTP nick-end labeling staining

Terminal deoxynucleotidyl transferase (TdT)-mediated dUTP nick-end labeling (TUNEL) staining was conducted with an in situ Apoptosis Detection Kit (Takara, Osaka, Japan) according to the manufacturer’s instruction.

### Western blotting

Retina-RPE choroids were homogenized with homogenate buffer, and the protein concentration was measured according to the methods previously reported [23]. Sodium dodecyl sulfate–PAGE was performed with 10% acrylamide. Separated proteins were transferred to the polyvinylidene difluoride membrane (Bio-Rad, Hercules, CA), and M-opsin was detected as previously described [23]. Briefly, the membrane was blocked for 1 h at room temperature with 1% normal goat or rabbit (for S-opsin only) serum in 0.05% Tween-20 in phosphate-buffered saline (Tw-PBS; 137 M NaCl, 10 M phosphate, 2.7 M KCl and a pH 7.4).abbit anti-L/M-opsin antibody (described as anti-M-opsin antibody; 0.2 µg/ml; Chemicon International, Temecula, CA), goat antiblue sensitive opsin antibody (described as anti-S-opsin antibody; 0.04 µg/ml; Santa Cruz Biotechnology, Delaware, CA), mouse antirhodopsin antibody (0.02 µg/ml; Chemicon International), rabbit anti-GNAT2 (cone transducin α) antibody (0.2 µg/ml; Abcam, Cambridge, MA), or rabbit anti-GNAT1 (rod transducin α) antibody (0.5 µg/ml; Abcam) was used as the primary antibody, respectively, and the membranes were incubated with diluted antibody overnight at 4 °C. After the membranes were washed three times with Tw-PBS, horseradish peroxidase (HRP)-conjugated goat antirabbit immunoglobulin G (IgG) (0.05 µg/ml; Dako, Cambridge, UK) or rabbit antigoat IgG (for detecting S-opsin; 0.05 µg/ml; Dako) was used as the secondary antibody, and the membranes were incubated at room temperature for 1 h. Immunoreactive signals were developed with ECL western blotting detection reagents (GE Healthcare, Piscataway, NJ), and the immunoreactive bands were captured with LAS3000 mini (Fujifilm, Tokyo, Japan). For detecting bands of glyceraldehyde-3-phosphate dehydrogenase (GAPDH) and β-actin, polyvinylidene difluoride membranes were reblotted with the ReBlot Western Blot Recycling Kit (Chemicon International), and rabbit anti-GAPDH antibody (0.02 µg/ml; Abcam) or mouse anti-β-actin antibody (0.1 µg/ml; Abcam) was used as the primary antibody. The integrated density of each band was quantified by using ImageJ software.

### Immunohistochemistry

Sections were rinsed with PBS and were quenched of endogenous peroxidase activity with 0.3% H_2_O_2_ in methanol for 15 min at room temperature to minimize background. After being washed with Tw-PBS, sections were blocked with 5% skim milk in Tw-PBS for 2 h. Rabbit anti-M-opsin antibody was diluted in blocking buffer (1:4,000) and applied overnight at 4 °C. Sections were then washed in Tw-PBS and treated with HRP-conjugated goat antirabbit IgG (1:500; Dako) overnight at 4 °C. After being washed with Tw-PBS, sections were incubated with tetrametylrhodamine-labeled tyramide (PerkinElmer Inc., Waltham, MA) for 10 min at room temperature. Following this procedure, sections were washed with Tw-PBS and mounted with Vectashield (Vector, Burlingame, CA) containing 4’-6’-diamidino-2-phenylindole (DAPI) to stain nuclei. Micrographs were taken from retinas randomly at 200× magnification, and the area of immunoreactivity with anti-M-opsin antibody in six regions of section was quantified using ImageJ software.

### Quantitative reverse-transcriptase polymerase chain reaction

Retina-RPE choroids were isolated, and total RNA was extracted from them with Isogen (Nippon Gene, Osaka, Japan). Then, cDNA were generated as described previously [23]. Briefly, RNA (1 µg each) was used to generate first strand cDNA with a first-strand cDNA Synthesis kit for RT–PCR (Roche Applied Science, Indianapolis, IN). PCR amplifications were conducted using SYBR Premix Ex taq (Takara) and 0.2 μM of each forward and reverse primer. Primers to amplify genes were as follows: M-opsin (forward: 5′-CTC TGC TAC CTC CAA GTG TGG-3′, reverse: 5′-AAG TAT AGG GTC CCC AGC AGA-3′); S-opsin (forward: 5′-TGT ACA TGG TCA ACA ATC GGA-3′, reverse: 5′-ACA CCA TCT CCA GAA TGC AAG-3′); rhodopsin (forward: 5′-CAA GAA TCC ACT GGG AGA TGA-3′, reverse: 5′-GTG TGT GGG GAC AGG AGA CT-3′); guanine nucleotide binding protein cone-specific transducin α-subunit (*GNAT2*; forward: 5′-GCA TCA GTG CTG AGG ACA AA-3′, reverse: 5′-CTA GGC ACT CTT CGG GTG AG-3′); *GNAT1* (forward: 5′-GAG GAT GCT GAG AAG GAT GC-3′, reverse: 5′-TGA ATG TTG AGC GTG GTC AT-3′), and β-actin (forward: 5′-GCT ACA GCT TCA CCA CCA CA-3′, reverse: 5′-TCT CCA GGG AGG AAG AGG AT-3′) [11,23]. Real-time RT–PCR reaction and normalization with β-actin were performed using previously published methods [23]. Briefly, reaction was performed under the fo;;owing cycling conditions: 95 °C for 10 s; and 26–40 cycles of 95 °C for 5 s and 60 °C for 20 s with LightCycler (Roche Applied Science). The specificity of amplication reactions was confirmed by melting-curve analysis and amplified PCR fragments were electrophoresed in a 1.5% agarose gel and visualized by ethidium bromide. A dilution series of cDNA samples was used to generate a standard curve and quantitative values of target genes were obtained with LightCycler software according to the manual. Expression levels of target genes were normalized by β-actin level in each sample.

### Statistical analyses

Statistical analyses of the target protein and gene expression were performed with an unpaired Student *t* test.

## Results

### The effect of explant culture on wild-type retina

**Figure 1:**
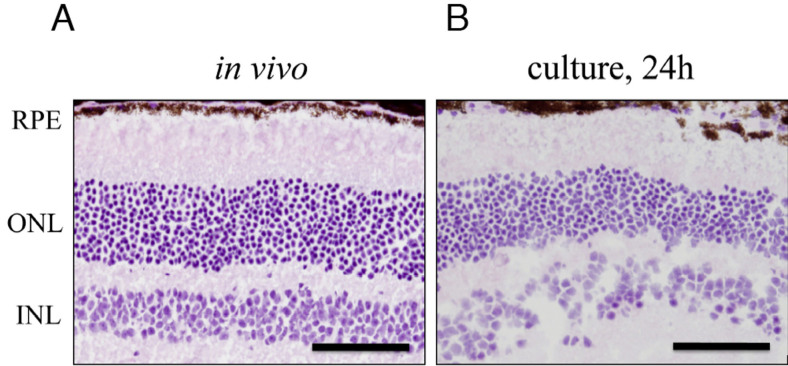
Histologic analysis of wild-type retina in vivo and after explant culture. The cryosections of wild-type retina in vivo (**A**) and for 24 h incubation after explant culture (**B**) were stained with hematoxylin and eosin. RPE, retinal pigment epithelium; ONL, outer nuclear layer; INL, inner nuclear layer. Scale bars=100 μm.

**Figure 2:**
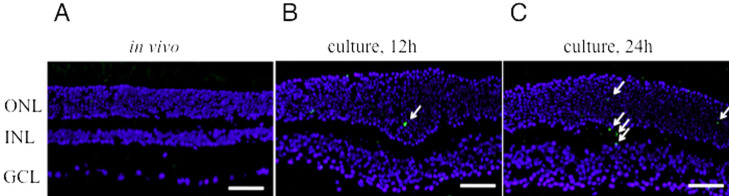
Detection of apoptotic cells with TUNEL staining. Wild-type retina in vivo as negative control (**A**), explant culture of the wild-type retina for 12 h (**B**), and 24 h incubation (**C**). *Blue*: nuclear staining with DAPI. *Green*: TUNEL-positive staining indicated by arrows. ONL, outer nuclear layer; INL, inner nuclear layer; GCL, ganglion cell layer. Scale bars=100 μm.

We analyzed retinal sections to evaluate the explant culture method. Eyes of wild-type (C57BL/6) mice were cultured and stained with hematoxylin and eosin. After 24 h of explant culture, the outer retinal structure was relatively preserved (Figure 1). In addition, TUNEL staining was performed to investigate whether apoptosis was induced by the explant culture itself in the retina. In the wild-type mice retina, TUNEL-positive cells were only rarely visible after 12 h and 24 h of explant culture (Figure 2).

### Level of M-opsin protein in the presence of a protease inhibitor mixture in *Rpe65*^−/−^ mice retina

**Figure 3:**
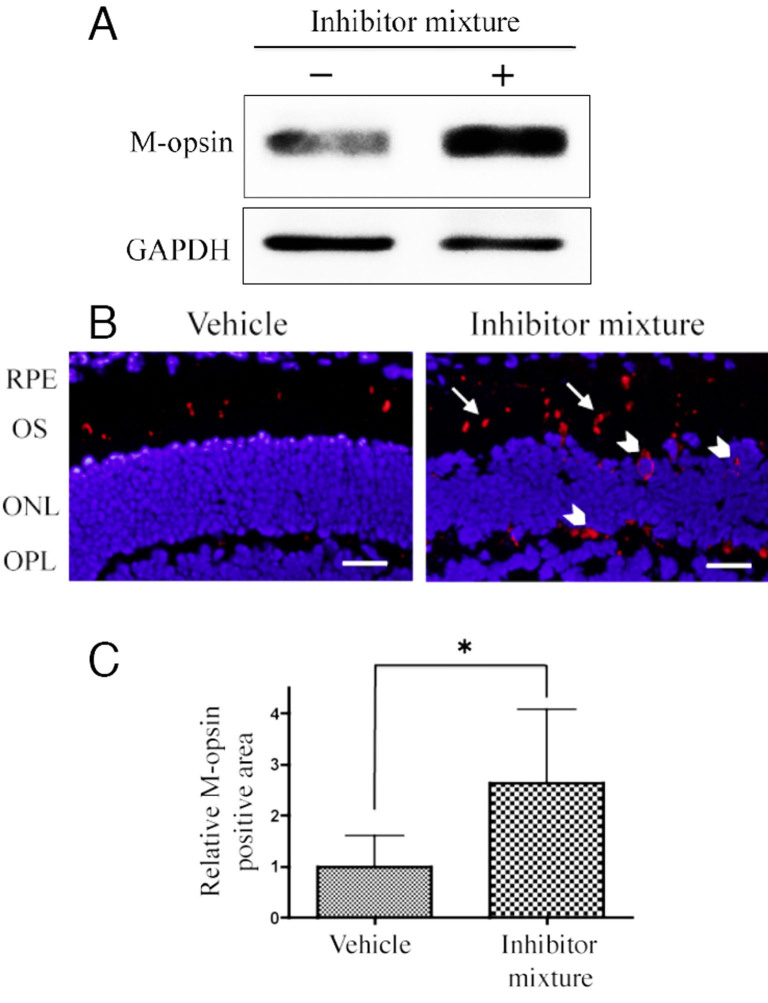
Western blotting and immunohistochemistry analysis of M-opsin expression treated with a protease inhibitor mixture (20 μM pepstatin A, 30 μM E64d, and 10 μM MG-132) for 24 h in *Rpe65*^−/−^ mice in retinal explants. **A**: Immunoblots were performed using retina-RPE choroids treated with the protease inhibitor mixture. A loading control with GAPDH was included for each immunoblot. **B**: Immunolocalization of M-opsin protein in the retina treated with a protease inhibitor mixture. M-opsin was stained with polyclonal anti-M-opsin antibody (red), and cell nuclei were labeled with DNA-binding dye DAPI (blue). The number of immunoreactive products increased in the cone outer segments (arrows), ONL, and OPL (arrowheads). **C**: Histograms showing the area of immunoreactivity of M-opsin in a 400-μm-wide section of the retina. Data are expressed as the mean±SD (n=6). *p<0.05. RPE, retinal pigment epithelium; OS, outer segment; ONL, outer nuclear layer; OPL, outer plexiform layer. Scale bars, 50 μm.

The amount of M-opsin protein in the retina treated with a protease inhibitor mixture (20 μM pepstatin A, 30 μM E64d, and 10 μM MG-132) was higher than that of M-opsin in the control retina for 24 h in *Rpe65*^−/−^ mice (Figure 3A). M-opsin protein was mainly localized to cone outer segments (COS) by vehicle treatment for 24 h in *Rpe65*^−/−^ mice. In contrast, M-opsin protein was found in cone outer segments, the outer nuclear layer (ONL), and the outer plexiform layer (OPL) in *Rpe65*^−/−^ mouse eyes treated with the protease inhibitor mixture for 24 h (Figure 3B). M-opsin-positive cells increased in cone outer segments (arrows) and notably appeared in the ONL and the OPL (arrowheads) with 24-h treatment with the protease inhibitor mixture. Immunohistochemistry revealed the area of immunoreactivity with anti-M-opsin antibody was significantly increased in retinas treated with the protease inhibitor mixture compared to control (an approximate 2.6 fold difference, p=0.0273, Figure 3C).

### Levels of M-opsin protein with the treatment of protease inhibitors in *Rpe65*^−/−^ mice retina

**Figure 4:**
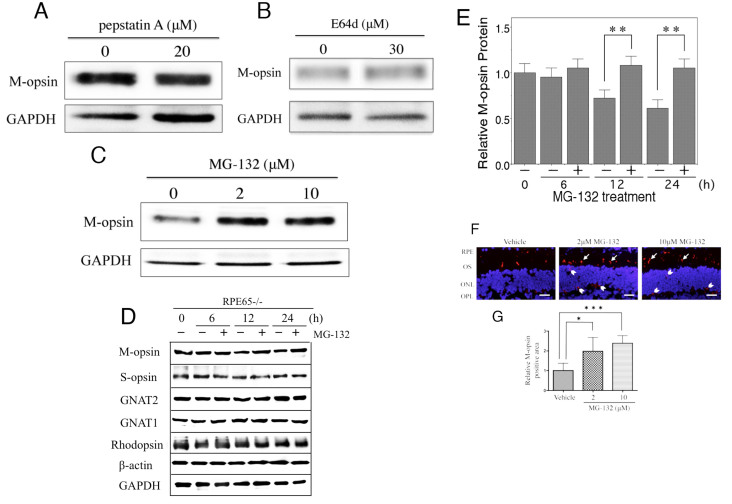
Expression of M-opsin protein in the retina after treatment with several types of protease inhibitors for 24 h in retinal explants. M-opsin protein levels were analyzed with western blotting after treatment with (**A**) pepstatin A, (**B**) E64d, and (**C**) MG-132. **D**: Effect of MG-132 on M-opsin, S-opsin, GNAT2, GNAT1, and rhodopsin at the protein level analyzed with western blotting. **E**: Quantitative analysis of M-opsin protein level. **F**: Immunohistochemistry in MG-132-treated retinas for 24 h showed M-opsin (red). Cell nuclei were contrasted with DAPI (blue). M-opsin-positive cells increased after treatment with 2 μM or 10 μM MG-132 compared with vehicle in cone outer segments (arrows), ONL, and OPL (arrowheads) in the retina. **G**: Densitometric analysis of immunofluorescence probed anti-M-opsin antibody in a 400-μm-wide section of retina. Histograms indicate mean±SD (n=6). *p<0.05, **p<0.01, ***p<0.001. RPE, retinal pigment epithelium; OS, outer segment; ONL, outer nuclear layer; OPL, outer plexiform layer. Scale bars, 50 μm.

To investigate which protease inhibitor was most effective on M-opsin degradation, protease inhibitors were used independently to treat eyes of *Rpe65*^−/−^ mice. Treatment with 20 μM pepstatin A or 30 μM E64d for 24 h did not change the level of M-opsin protein compared with vehicle treatment (Figure 4A,B). However, 2 μM or 10 μM MG-132 treatment for 24 h increased the amount of M-opsin protein compared with control (Figure 4C). We then tried to evaluate the effect of MG-132 on the protein level of not only M-opsin but also other photoreceptor specific proteins including S-opsin, rhodopsin, GNAT1, and GNAT2, respectively. Results showed that MG-132 specifically and significantly (p<0.01) inhibited downregulation of M-opsin at the protein level compared with vehicle treatment while the S-opsin, rhodopsin, GNAT1, and GNAT2 levels were not different between MG-132 and vehicle (Figure 4D,E). MG-132 treatment also increased M-opsin-expressing cells in the area of cone outer segments (arrows), the ONL, and the OPL (arrowheads; Figure 4F). Quantitative analysis was performed on the immunohistochemistry; the area of M-opsin immunoreactivity was significantly increased by treating with 2 μM MG-132 (approximately 2.0 fold, p=0.0108) and 10 μM MG-132 (approximately 2.4 fold, p<0.0001) compared to the control, respectively (Figure 4G).

### The effect of 9-*cis*-retinal treatment on M-opsin protein expression in *Rpe65*^−/−^ mice

**Figure 5:**
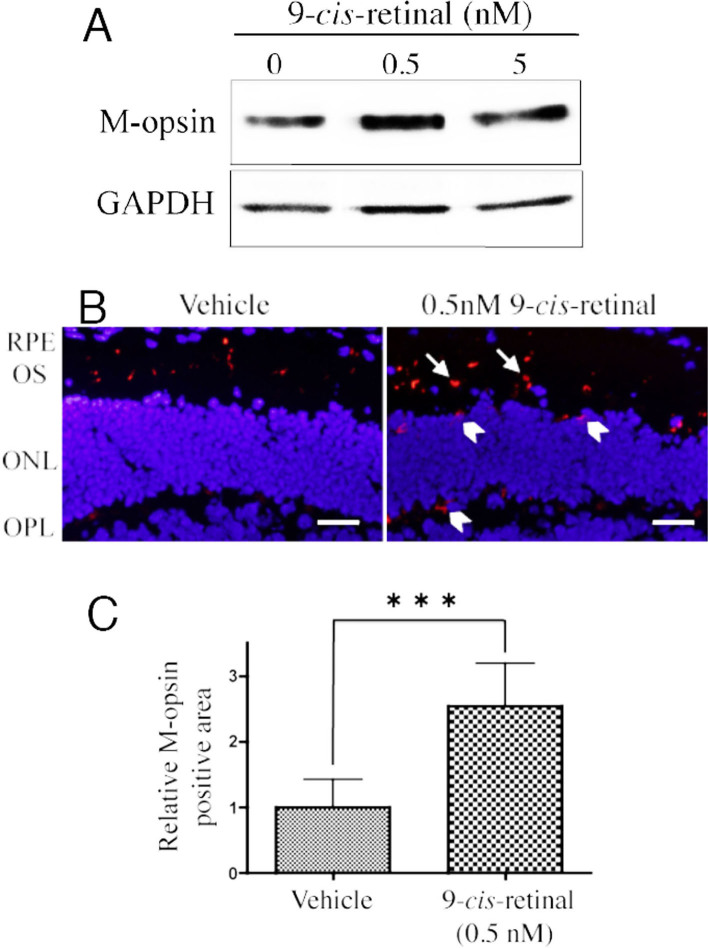
Effect of 9-*cis*-retinal on M-opsin expression in *Rpe65*^−/−^ mice in retinal explants. **A**: Retina-RPE choroids incubated with 0.5 nM or 5 nM 9-*cis*-retinal for 24 h were used for western blotting to detect M-opsin protein. **B**: Immunolocalization of M-opsin treated 0.5 nM 9-*cis*-retinal for 24 h in the *Rpe65*^−/−^ mice retina. Immunoreactivity of M-opsin (red) was higher with 0.5 nM 9-*cis*-retinal treatment compared to vehicle treatment in the cone outer segments (arrows), ONL, and OPL (arrowheads) in the retina. **C**: Histological findings of the density on the reactions labeled anti-M-opsin antibody in a 400-μm-wide section of the retina. Values represented mean ± SD (n=6). ***p<0.001. RPE, retinal pigment epithelium; OS, outer segment; ONL, outer nuclear layer; OPL, outer plexiform layer. Scale bars, 50 μm.

To determine whether the degradation of the M-opsin protein was caused by depletion of 11-*cis*-retinal in *Rpe65*^−/−^ mice, 9-*cis*-retinal was substituted for 11-*cis*-retinal. The levels of M-opsin protein expression increased in the *Rpe65*^−/−^ mice retinas treated with 0.5 nM 9-*cis*-retinal for 24 h (Figure 5A). High immunoreactivity to M-opsin with 0.5 nM 9-*cis*-retinal treatment compared to vehicle treatment was detected in cone outer segments (arrows), the ONL, and the OPL (arrowheads) with immunohistochemistry (Figure 5B). M-opsin protein was only slightly detectable in the ONL and the OPL of the vehicle-treated retinas, but M-opsin-positive cells were present in these areas with the treatment of 0.5 nM 9-*cis*-retinal. The signal intensity of the probed anti-M-opsin antibody was measured to determine quantity. Immunohistochemistry showed the area of M-opsin reactivity was significantly increased with treatment by 0.5 nM 9-*cis*-retinal; a 2.5 fold increase (p=0.0006) was seen relative to vehicle treatment (Figure 5C).

**Figure 6:**
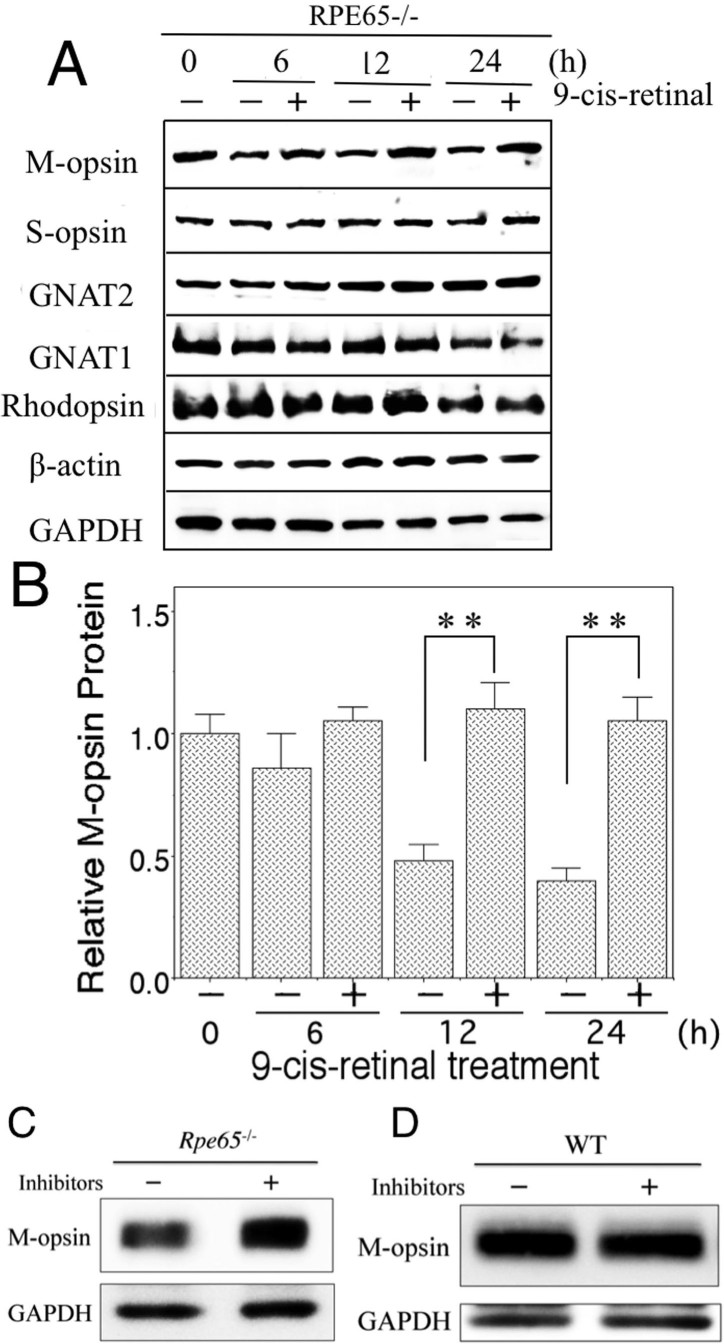
Time course of 9-*cis*-retinal treatment in *Rpe65*^−/−^ mice on photoreceptor specific proteins and the inhibitory effect of a protease inhibitor mixture on M-opsin degradation in *Rpe65*^−/−^ mice and wild-type mice at three weeks in retinal explants. **A**: The M-opsin, S-opsin, GNAT2, GNAT1, and rhodopsin levels treated with 0.5 nM 9-*cis*-retinal for 6, 12, or 24 h are shown with immunoblot in *Rpe65*^−/−^ mice. **B**: Quantitative analysis of effect of 9-*cis*-retinal on the M-opsin protein level. Histograms indicate mean±SD (n=6), **p<0.01. Western blots were performed to analyze the expression of M-opsin protein in the retina-RPE choroids treated with a protease inhibitor mixture (20 μM pepstatin A, 30 μM E64d, and 10μM MG-132) for 12 h in *Rpe65*^−/−^ mice (**C**) and wild-type mice (**D**).

Treatment with 0.5 nM 9-*cis*-retinal for 6 or 12 h also significantly increased the levels of M-opsin protein compared with control (p<0.01) while S-opsin, GNAT2, GNAT1, and rhodopsin did not show any difference at the protein level between 9-*cis*-retinal and vehicle treatment (Figure 6A,B). GAPDH western blots did not differ between 0.5 nM 9-*cis*-retinal treatment and vehicle treatment, but the slight decrease in the GAPDH protein levels depends on the culture time while the expression of β-actin showed constant levels during the culture time (Figure 6A). Treatment with a protease inhibitor mixture for 12 h increased the level of M-opsin protein in the retinas of *Rpe65*^−/−^ mice but not wild-type mice (Figure 6C,D).

### The effect of 9-*cis*-retinal on the expression of photoreceptor genes in *Rpe65*^−/−^ mice

**Figure 7:**
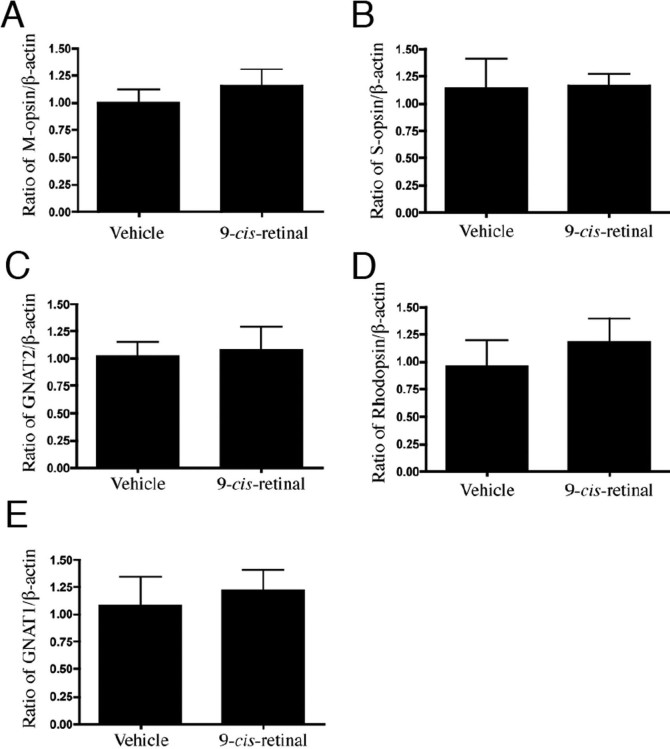
Effect of treatment with 9-*cis*-retinal for the expression of photoreceptor specific genes in *Rpe65*^−/−^ mice at three weeks. Semiquantitative real-time RT–PCR was performed to determine transcriptional levels of M-opsin (**A**), S-opsin (**B**), *GNAT2* (**C**), rhodopsin (**D**), and *GNAT1* (**E**). No significant change in the transcriptional levels of five genes was present between 0.5 nM 9-*cis*-retinal treatment and vehicle treatment for 12 h. Values represent mean±SD (n=6).

We used real-time RT–PCR analysis to evaluate transcriptional levels in retina-RPE choroids from *Rpe65*^−/−^ mice treated with 9-*cis*-retinal for 12 h. The relative amounts of cone photoreceptor specific gene (M-opsin, S-opsin and *GNAT2*; cone transducin α subunit) mRNAs were not different between vehicle and 0.5 nM 9-*cis*-retinal treatment (Figure 7A-C). The expression levels of mRNA of rod photoreceptor specific genes (rhodopsin and *GNAT1*; rod transducin α subunit) also did not differ significantly between 0.5 nM 9-*cis*-retinal treatment and vehicle treatment in *Rpe65*^−/−^ mice (Figure 7D,E).

## Discussion

Previously, we reported that the transcriptional level of M-opsin in *Rpe65*^−/−^ mice was not significantly different from that of wild-type mice at three or five weeks of age [23]. However, protein levels of M-opsin were dramatically decreased in *Rpe65*^−/−^ mice compared with wild-type mice [23]. These results indicate that M-opsin protein may degrade faster in *Rpe65*^−/−^ mice than in wild-type mice. Although Znoiko et al. [11] reported that M-opsin was transcriptionally downregulated in the Rpe65^−/−^ mice, they did not examine M-opsin expression at the protein level. In addition, their RT–PCR methods [11] were different from our previous reports [23]. The main differences include the quantitative analysis methods (their ΔΔCt method versus our standard curve analysis), reference genes (18S rRNA versus β-actin mRNA), and primers to generate cDNA (oligo dT versus random primers). These differences in RT–PCR methods may have produced different results. In the present study, to evaluate our hypothesis of the posttranslational degradation of M-opsin, we used explant cultures. The present study demonstrated that M-opsin protein in *Rpe65*^−/−^ mice degraded more than that of wild-type mice and that the degradation was specifically and significantly inhibited by MG-132 or 9-*cis*-retinal treatment. These results suggest that 11-*cis*-retinal contributes to the stability of M-opsin protein and that depletion of 11-*cis*-retinal induces the degradation of M-opsin protein via a proteasome pathway in the mouse retina. In our current experimental conditions, however, high immunoreactivity to M-opsin was detected not only in cone outer segments but also in the ONL and the OPL (Figure 5B). Possibilities that may explain these results are the concentration and/or duration of 9-*cis*-retinal treatment may not be enough to correct mis-trafficking of M-opsin, although 9-*cis*-retinal treatment partially suppresses M-opsin degradation, and the explant condition may not be optimized for protein trafficking rather than an in vivo condition. The latter possibility may also explain why MG-132 or 9-*cis*-retinal treatment did not increase the expression of GNAT2, one of peripheral membrane proteins in cone outer segments. In a previous study by Zhang et al. [12], administration of 11-*cis*-retinal corrected abnormal trafficking of GNAT2, and consequently increased its protein expression level. Their study suggests that not only cone opsins require 11-*cis*-retinal to fold correctly and to traffic to COS, but also other COS proteins may require cone opsins as guides to be cotargeted and targeted correctly. In the present study, however, administration of 9-*cis*-retinal did not increase the expression of GNAT2 at the protein level (Figure 6A). The present results suggest that COS proteins such as GNAT2 were degraded probably because they were not targeted to COS properly due to mis-trafficking of M-opsin. The amount of GNAT2 was not expected to increase if neither MG-132 nor 9-*cis*-retinal seemed to be able to correct the M-opsin mis-trafficking problem. In addition, because administration of 11-*cis*-retinal to *Rpe65*^−/−^*Rho*^−/−^ mice also only partially corrected mis-trafficking of M-opsin [12], trafficking of M-opsin to COS may require other factors besides 11-*cis*-retinal. Further study is needed to clarify these points.

Huber et al. [27] reported that chromophores did not regulate gene transcription of Rh1 opsin in *Drosophila*, but chromophore supply was important for stabilizing the Rh1 opsin protein. Previous studies have demonstrated that 11-*cis*-retinal regulated stability for rod opsin [20-22]. In addition, the mutant rhodopsin misfolded protein was degraded by proteasome, and improved folding and stability were obtained by adding chromophores [28-30]. These data suggest that chromophores act like chaperone molecules for the rod opsin protein. On the other hand, no study has reported that chromophores enhance stability of cone opsins as far as we understand. Our data indicate that chromophores play a role in maintaining the stable conformation of M-opsin protein.

Previous studies have reported that intraperitoneal injection of 11-*cis*-retinal can restore cone ERG responses in *Rpe65*^−/−^ mice and *Rpe65*^−/−^*Rho*^−/−^ mice [10,31]. These data suggest that available exogenous 11-*cis*-retinal may promote cone pigments in the mouse retina. Moreover, administration of 9-*cis*-retinal or 9-*cis*-retinyl acetate preserved ERG responses in various mice lacking 11-*cis*-retinal and in *Rpe65* mutant dogs [8,9,32-35]. These studies indicate that 9-*cis*-retinal is also useful as a chromophore, and visual pigments are generated by exogenous 9-*cis*-retinal. Our results showed that 9-*cis*-retinal treatment prevented degradation of M-opsin protein in *Rpe65*^−/−^ mice. These observations indicate that chromophore-free M-opsin pigments are degraded and endogenous 11-*cis*-retinal is critical for the stability of M-opsin protein in the mouse retina. Furthermore, the degradation of M-opsin protein is prevented when exogenous 9-*cis*-retinal is available to act as a chromophore. In the present study, 0.5 nM 9-*cis*-retinal was effective for preserving M-opsin protein in *Rpe65*^−/−^ mice to some extent (Figure 6A). Treatment of *GNAT1*^−/−^*Lrat*^−/−^ and *GNAT1*^−/−^*Rpe65*^−/−^ mouse eyes with 9-*cis*-retinyl acetate restored cone-specific ERG [32]. This recovery of cone ERG responses may be partially due to the inhibition of M-opsin protein degradation through 9-*cis*-retinyl acetate treatment.

We observed a tendency for GAPDH levels to slightly decrease in our explant culture from 6 to 24 h, although β-actin levels maintained the same expression level (Figures 4D and Figure 6A). These phenomena suggest that GAPDH may either be less stable than β-actin or GAPDH expression may be downregulated with the culture condition. These possibilities need to be investigated in further studies.

We previously reported that the transcriptional level of rhodopsin decreased in *Rpe65*^−/−^ mice aged three to seven weeks [23]. We concluded that 11-*cis*-retinal directly regulated the transcriptional level of rhodopsin, and thus the cause of downregulation of rhodopsin mRNA in *Rpe65*^−/−^ mice was depletion of 11-*cis*-retinal in the retina. To test this hypothesis in the current experiments, we evaluated the transcriptional level of rhodopsin with 9-*cis*-retinal treatment in *Rpe65*^−/−^ mice with real-time RT–PCR. The amount of rhodopsin mRNA expression in the 9-*cis*-retinal treated retinas was not significantly different from the control in *Rpe65*^−/−^ mice (Figure 7D). In addition, the transcriptional levels of other photoreceptor specific genes also were unchanged (Figure 7). These data demonstrate that the chromophore does not directly regulate the transcriptional expression of rhodopsin and other photoreceptor specific genes. Downregulation of rhodopsin mRNA expression in *Rpe65*^−/−^ mouse retinas might indicate damage or stress on rod photoreceptor cells.

Our data showed that the M-opsin protein level increased in *Rpe65*^−/−^ mice with MG-132 treatment, but not with pepstatin A or E64d treatment (Figure 4). Pepstatin A is an inhibitor of cathepsin D; E64d is an inhibitor of calpains and cathepsins B/H/L [36-41]. MG-132 is an inhibitor of proteasome, cathepsins, and calpains. Inhibition of the degradation of M-opsin protein in *Rpe65*^−/−^ mice with MG-132 treatment implies that the M-opsin protein is degraded by proteasome and/or cathepsins and/or calpains. Since pepstatin A and E64d did not inhibit the degradation of M-opsin protein, the degradation was not due to cathepsins and calpains. Briefly, these data indicate that the M-opsin protein in the *Rpe65*^−/−^ mouse retina may be degraded via the proteasome pathway rather than by lysosomal enzymes. The proteasome system is required for maintaining biologic homeostasis and regulating many crucial processes in cells [42]. Ubiquitinated proteins are degraded as substrates by 26S proteasome, an adenosine-5′-triphosphate-dependent proteolytic complex [43]. The 26S proteasome degrades misfolded proteins, various cell cycle regulators, transcription factors, and tumor suppressors [44,45]. M-opsin lacking 11-*cis*-retinal may be a misfolding protein in *Rpe65*^−/−^ mice, and this misfolding might trigger degradation of M-opsin protein by the proteasome system. Zhang et al. reported that M-opsin in *Lrat*^−/−^ mice was only transiently ubiquitinated before being degraded by the proteasome [24]. Their results may also suggest the possibility that the ubiquitinated M-opsin is quickly degraded by the proteasome system.

In the retina, α-crystallin is expressed and classified as a member of the heat shock protein family of molecular chaperones. Deretic et al. [46] reported that α-crystallin is associated with post-Golgi membranes concurrently with newly synthesized rhodopsin in frog photoreceptors. These data suggest that α-crystallin might play a role in correct folding of rhodopsin on post-Golgi membranes. In addition, Griciuc et al. [47] previously reported the possibility that misfolded rhodopsin^P23H^ required the endoplasmic reticulum associated degradation pathway to be degraded by the proteasome [48]. In the mouse retina, M-opsin protein might also regulate folding and stability by chromophores on post-Golgi membranes. We presume that the misfolded M-opsin is exported from the ER to the cytoplasm by the endoplasmic reticulum associated degradation pathway for destruction by the proteasome in the cytoplasm in *Rpe65*^−/−^ mice.

In summary, we conclude that M-opsin protein may be degraded via the proteasome pathway, and the degradation was inhibited with 9-*cis*-retinal treatment in the *Rpe65*^−/−^ mice retina to some extent. This study demonstrated that chromophore-free M-opsin is degraded quickly and chromophores have a crucial role in M-opsin stabilization as the ligands in the mouse retina.
